# The complete chloroplast genome sequence of the medicinal plant *Siegesbeckia orientalis* L., the first in the genus *Siegesbeckia*

**DOI:** 10.1080/23802359.2019.1698355

**Published:** 2019-12-11

**Authors:** Jingting Liu, Mei Jiang, Liqiang Wang, Linfang Huang, Haimei Chen, Chang Liu

**Affiliations:** Key Laboratory of Bioactive Substances and Resource Utilization of Chinese Herbal Medicine from Ministry of Education, Institute of Medicinal Plant Development, Chinese Academy of Medical Sciences and Peking Union Medical College, Beijing, P. R. China

**Keywords:** Chloroplast genome, *Siegesbeckia orientalis* L., Photosynthesis

## Abstract

*Siegesbeckia orientalis* L. is a plant with important medicinal and economic values. We reported the first complete chloroplast genome sequence of *S. orientalis*. This genome is 151,821 bp in length and comprises a large single-copy region of 83,540 bp, a small single-copy region of 18,225 bp and a pair of inverted repeat regions of 25,028 bp each. It encodes 138 genes, including 79 protein-coding genes, 27 tRNA genes, and 4 rRNA genes. The phylogenomic analysis showed that *S. orientalis* and *Guizotia abyssinia* were clustered together. This genomewill lay the foundation for the molecular discovery and phylogenomic study of this genus.

Siegesbeckiae Herba is a medicinal material listed in Chinese Pharmacopeia used against rheumatism, arthritis, hypertension, malaria, and neurasthenia (Pharmacopoeia Commission of People’s Republic of China 2015). it derives from the aerial part of three plants: *Siegesbeckia orientalis* L., *Siegesbeckia pubescens* Makino, and *Siegesbeckia glabrescens* Makino (Tao et al. 2018). Previously, the ITS1-5.8S-ITS2 regions have been sequenced to discriminate the three species. However, across the total sequences of 730–732 bp, the three plants shared 99.27% similarity with each other (Gao et al. 2018) and were difficult to distinguish. Molecular markers with higher resolution are in urgent need to support the discrimination of *S. orientalis* and its adulterant species as well as molecular breeding studies of *S. orientalis.* In this study, we have sequenced and analyzed the complete chloroplast genome of *S. orientalis* as the first step for marker discovery.

Fresh leaves were collected from the Central China Medicinal Botanical Garden, EnShi, China (E30°17′71′′, N109°74′38′′) and identified as from *S. orientalis* by Professor Zhao Zhang. Genomic DNA was extracted from the leaves with a plant genomic DNA kit (Tiangen Biotech, China), isolated and subjected to Next Generation DNA sequencing (NGS) using the Hiseq 2500 platform (Illumina, San Diego, CA). The raw sequence data were assembled into a single contig corresponding to the chloroplast genome by using NOVOPlasty (v. 2.7.2) (Dierckxsens et al. 2017). CpGAVAS2 was used to annotate the chloroplast genome (Shi et al. 2019). A voucher specimen and its DNA were deposited at Institute of Medicinal Plant Development (Accession number: 201808168).

The chloroplast genome of *S. orientalis* (GenBank accession number: MN240004) is 151,821 bp in size with a pair of inverted repeat (IR) regions of 25,028 bp separated by a large single-copy (LSC) region of 83,540 bp and a small single-copy (SSC) region of 18,225 bp. The chloroplast genome encodes 138 genes, including 79 protein-coding genes, 27 tRNA genes, and 4 ribosome rRNA genes. Among them, seven genes contain one intron and two genes contain two introns. Furthermore, six tRNA genes contain one intron. The length of the protein-coding sequence (CDS) of the chloroplast genome is 77,922 bp, accounting for 51.32% of the whole genome. The lengths of the rRNA and tRNA genes were 9394 and 2655 bp, which were 6.19 and 1.75% of the total length, respectively. To examine the phylogenetic position of *S. orientalis*, we constructed the phylogenetic tree between *S. orientalis* and 11 other closely related species, using feature frequency profiles (FFP) method (Sims and Kim 2011) implemented in PlasDB software (http://www.herbalgenomics.org/plasdb) based on whole chloroplast genome sequences. The results showed that *S. orientalis* and *Guizotia abyssinia* were clustered together with a support value of 100 (Figure 1), which is consistent with those from the BLAST analysis results (not shown). The identification and characterization of the complete chloroplast genome of *S. orientalis* will help to identify the genes associated with the production of its bioactive compounds and to understand the evolutionary relationship of species in this genus.

**Figure 1.**
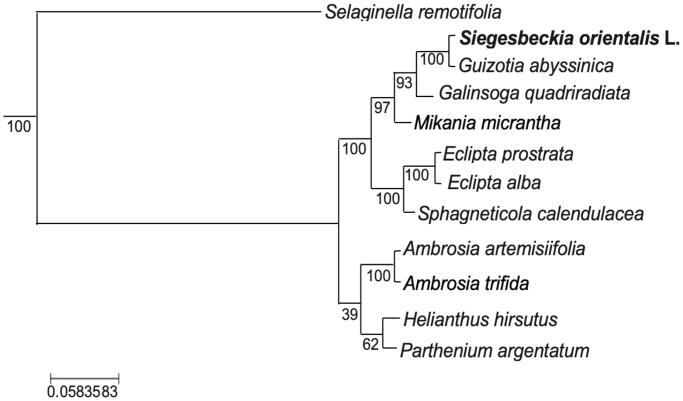
The phylogenetic tree constructed with the ‘feature frequency profiles’ method implemented in PlasDB for *Siegesbeckia orientalis* and its closest relatives using complete chloroplast genome sequences. The number represents the bootstrap values for the corresponding branch. *Selaginella remotifolia* was used as the outgroup. The Genbank accession numbers for the species are: *Selaginella remotifolia* (NC_041644.1), *Guizotia abyssinica* (NC_010601.1), *Galinsoga quadriradiata* (NC_031853.1), *Mikania micrantha* (NC_031833.1), *Eclipta prostrata* (NC_030773.1), *Eclipta alba* (NC_039774.1), *Sphagneticola calendulacea* (NC_039346.1), *Ambrosia artemisiifolia* (NC_035875.1), *Ambrosia trifida* (NC_036810.2), *Helianthus hirsutus* (NC_023111.1), and *Parthenium argentatum* (NC_013553.1).
